# Acquired tumor cell resistance to sunitinib causes resistance in a HT-29 human colon cancer xenograft mouse model without affecting sunitinib biodistribution or the tumor microvasculature

**DOI:** 10.18632/oncoscience.106

**Published:** 2014-12-15

**Authors:** Kristy J. Gotink, Henk J. Broxterman, Richard J. Honeywell, Henk Dekker, Richard R. de Haas, Kiersten M. Miles, Remi Adelaiye, Arjan W. Griffioen, Godefridus J. Peters, Roberto Pili, Henk M.W. Verheul

**Affiliations:** ^1^ Department of Medical Oncology, VU University Medical Center, Amsterdam, The Netherlands; ^2^ Department of Medicine, Genitourinary Section, Roswell Park Cancer Institute, Buffalo, NY, USA

**Keywords:** Sunitinib, resistance, lysosomes, angiogenesis, host-factors

## Abstract

Acquired resistance to anti-angiogenic tyrosine kinase inhibitors is an important clinical problem in treating various cancers. To what extent acquired resistance is determined by microenvironmental host-factors or by tumor cells directly is unknown. We previously found that tumor cells can become resistant to sunitinib *in vitro.* Here, we studied to what extent *in vitro* induced resistance of tumor cells determines *in vivo* resistance to sunitinib. In severe combined immunodeficient mice, tumors were established from HT-29 parental colon cancer cells (HT-29PAR) or the *in vitro* induced sunitinib resistant HT-29 cells (HT-29SUN). Treatment with sunitinib (40mg/kg/day) inhibited tumor growth of HT-29PAR tumors by 71±5%, while no inhibition of HT-29SUN tumor growth was observed. Intratumoral sunitinib concentrations and reduced MVD were similar in both groups. Ki67 staining revealed that tumor cell proliferation was significantly reduced with 30% in HT-29PAR tumors, but unaffected in HT-29SUN tumors upon sunitinib treatment. The lysosomal capacity reflected by LAMP-1 and -2 expression was higher in HT-29SUN compared to HT-29PAR tumors indicating an increased sequestration of sunitinib in lysosomes of resistant tumors. In conclusion, we demonstrate that tumor cells rather than host-factors may play a crucial role in acquired resistance to sunitinib *in vivo.*

## INTRODUCTION

Multiple anti-angiogenic tyrosine kinase inhibitors (TKIs) have been approved for the treatment of patients with disseminated cancers. Acquired resistance to these type of agents is an important clinical problem in treating patients with various types of cancer. In general, protein kinase inhibitors are effective in multiple different cancer types, but resistance inevitably emerges. Acquired mechanisms of resistance to kinase inhibitors include reactivation of the target through a secondary mutation, activation of upstream or downstream effectors, activation of a bypass oncoprotein and microenvironmental factors [1].

One of these TKIs, sunitinib, is currently approved for treatment of patients with gastrointestinal stroma tumors (GIST) refractory to imatinib, advanced pancreatic neuroendocrine tumors (pNET) and metastasized renal cell carcinoma (RCC) [2-4]. For other tumor types, sunitinib showed some clinical activity in a subgroup of patients, for example in colorectal cancer [5]. Sunitinib has been developed as an anti-angiogenic agent primarily targeting the vascular endothelial growth factor receptors (VEGFRs) and platelet-derived growth factor receptors (PDGFRs) [6]. Sunitinib also inhibits other kinases with higher or lower affinity that are active in endothelial cells and pericytes as well as in tumor cells [7, 8]. The inhibition of these kinases might be important for its antitumor activity as well.

Several potential mechanisms that contribute to sunitinib resistance include the induction of alternative angiogenic growth factors [9], epithelial to mesenchymal transformation of the tumor microenvironment [10], alternative bone marrow support [11,12] or altered pharmacokinetics, for example an increased metabolism of sunitinib, altered drug distribution, increased cellular efflux or inhibited drug uptake [13, 14]. Still, the underlying mechanism(s) to fully explain and overcome acquired resistance to sunitinib remain(s) unclear. Most studies have been focusing on potential angiogenic-factor-mediated mechanisms of resistance and the microenvironment of the host [1;9-12;15-17], while relatively little attention has been paid to the contribution of tumor-cell-related mechanisms of resistance to sunitinib, with the exception for sunitinib resistance in c-KIT expressing GIST and acquired resistance to sunitinib by tumor cell expression of the extracellular matrix metalloproteinase inducer [18;19]. Therefore, it is important to have a more complete understanding of all possible mechanisms contributing to sunitinib resistance in order to develop new treatment strategies to overcome this resistance.

Previously, we reported that tumor cells are sensitive to sunitinib treatment *in vitro* at clinically relevant intratumoral sunitinib concentrations [20]. These findings indicate that sunitinib directly inhibit tumor cell growth rather than only inhibiting angiogenesis. In addition, when cultured *in vitro*, we were able to induce tumor cell resistance to sunitinib in several cell lines upon continuous exposure to increasing doses of sunitinib. This acquired tumor cell resistance was transient and not related to genetic alterations. The underlying mechanism of this resistance was related to an increased lysosomal sequestration of sunitinib limiting its intracellular antitumor activity. Based on these findings we hypothesized that acquired tumor cell resistance to sunitinib contributes to the development of resistance to sunitinib in patients. Therefore, we studied here to what extent tumor cells may contribute to sunitinib resistance *in vivo*. We compared growth properties of *in vitro* sunitinib resistant HT-29 colon cancer cells with the parental HT-29 cells in a murine model and determined their *in vivo* sensitivity to sunitinib treatment. We show that tumor cells rather than host factors play a crucial role in acquired resistance to sunitinib *in vivo* based on comparable intratumoral sunitinib concentrations and reduced microvessel density in both groups, while tumor cell proliferation was only reduced in parental tumors and lysosomal capacity was increased in resistant tumors. In addition, we examined whether chloroquine, a clinical available antimalarial drug, which was recently shown to potentiate antitumor activity of sunitinib [21] and inhibits lysosomal function [22-24] could revert sunitinib resistance in this *in vivo* model.

## RESULTS

### *In vivo* growth and sunitinib treatment of HT-29 parental and sunitinib resistant cells

Prior to *in vivo* experiments, *in vitro* resistance to sunitinib was confirmed similar to our previous report [20]. Although both sensitive and resistant 786-O and HT-29 cells were injected in mice, reliable solid tumours from 786-OPAR and 786-OSUN cells in mice in 3 independent experiments did not form. Because, the IC_50_ values for sunitinib in 786-OPAR and 786-OSUN cells were in the same range as in HT-29PAR and HT-29SUN cells, respectively [20], we continued the *in vivo* experiments with HT29 xenografts.

**Figure 1:**
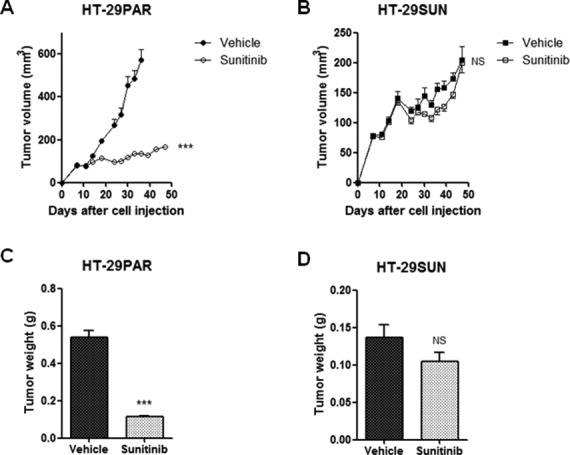
Sunitinib treatment of HT-29PAR and HT-29SUN tumors (A) and (B) Growth curves of tumors established from HT-29 parental (HT-29PAR) (A) and HT-29 sunitinib resistant (HT-29SUN) (B) tumor cells after tumor cell injection (5×10^6^ cells; n = 6). Mice received treatment with vehicle or sunitinib malate (40 mg/kg/day). (C) and (D) Tumor weights at the end of the experiment of HT-29PAR (C) and HT-29SUN (D) tumors. Results are shown as mean ± SEM (n = 6); ***, *P* < 0.001, NS= not significant.

Seven days after injection of HT-29PAR and HT-29SUN tumor cells, tumors were established *in vivo* (50-100 mm^3^ in size) and treatment with sunitinib malate (40 mg/kg/day) or vehicle was started. Vehicle-treated mice carrying tumors established from HT-29PAR cells were sacrificed after 30 days of treatment, because of ulceration and size. Sunitinib significantly inhibited the growth rate of HT-29PAR tumors with 76 ± 1% (mean ± SEM, n = 6; *P* < 0.001; Figure 1*A*), while growth of HT-29SUN tumors was unaffected after 40 days of sunitinib treatment (2 ± 8% inhibition, *P* = ns (not significant); Figure 1*B*). Similar, the weight of sunitinib-treated HT-29PAR tumors was lower than vehicle-treated HT-29PAR tumors, with weights of 0.11 ± 0.01 g versus 0.54 ± 0.04 g, respectively (*P* < 0.001; Figure 1*C*). No significant difference was observed between the weights of sunitinib- and vehicle-treated HT29-SUN tumors (0.10 ± 0.01 versus 0.14 ± 0.02 g, *P* = ns; Figure 1*D*).

In a second experiment, in which tumors were established by transplantation of tumor pieces from other mice, these results were confirmed (Figure S1). In both *in vivo* experiments, the mice tolerated sunitinib treatment very well with a maximal weight loss in an individual mouse of less than 10%.

### Intratumoral sunitinib concentrations

**Figure 2:**
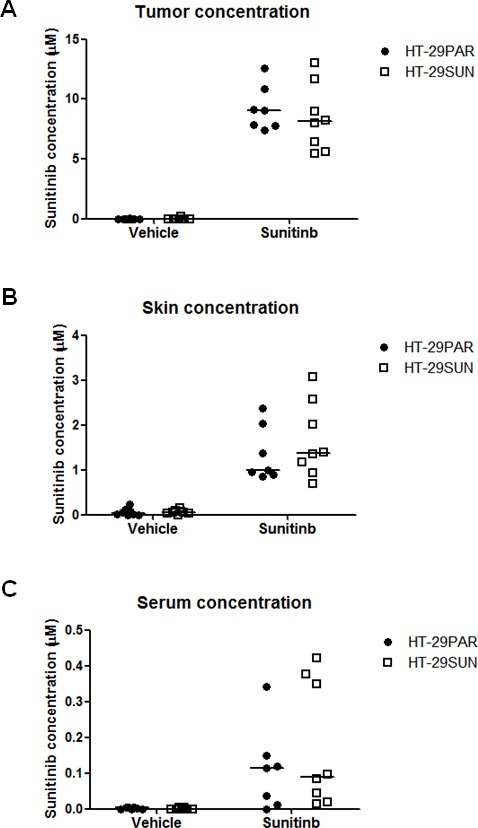
Sunitinib concentrations in tumors, skin and serum Sunitinib concentrations at the end of the experiment in mice bearing HT-29 parental (HT-29PAR) or HT-29 sunitinib resistant (HT-29SUN) tumors. Sunitinib concentrations intratumoral (A), in skin (B) and in serum (C) were measured with liquid chromatography - tandem mass spectrometry (LC-MS/MS). Results are shown as individual mice (n = 8) with median indicated.

Intratumoral sunitinib concentrations were determined in mice at the end of treatment. In sunitinib-treated tumors, intratumoral concentrations were comparable between HT-29PAR and HT-29SUN tumors with 9.1 μM (7.4 – 12.6 μM) and 8.1 μM (5.5 – 13.1 μM) (median (range); n = 8; *P* = ns), respectively (Figure 2*A*). In addition, sunitinib concentrations in normal skin tissues of these mice were comparable between mice bearing HT-29PAR and HT-29SUN tumors (1.0 (0.9 – 2.4) μM versus 1.4 (0.7 – 3.1) μM sunitinib (*P* = ns; Figure 2*B*)). The corresponding intratumoral / skin sunitinib concentrations in micrograms sunitinib per gram tissue are shown in table 1. Sunitinib serum concentrations varied largely between mice, but were comparable between mice bearing HT-29PAR and HT29-SUN tumors: 0.1 μM (non-detectable – 0.3 μM) versus 0.1 μM (0.0 – 0.4 μM) sunitinib (*P* = ns; Figure 2*C*).

**Table 1:** Sunitinib concentrations (median (range)) in tumor and in normal skin

		micromole (μM)	μg per gram tissue (μg / g)
Tumor	HT-29PAR HT-29SUN	9.1 (7.4 – 12.6) 8.1 (5.5 – 13.1)	3.6 (3.0 – 5.0) 3.2 (2.2 – 5.2)
Skin	HT-29PAR HT-29SUN	1.0 (0.9 – 2.4) 1.4 (0.7 – 3.1)	0.4 (0.3 – 0.9) 0.5 (0.3 – 1.2)

### Microvessel density, tumor cell proliferation and lysosomal capacity

**Figure 3:**
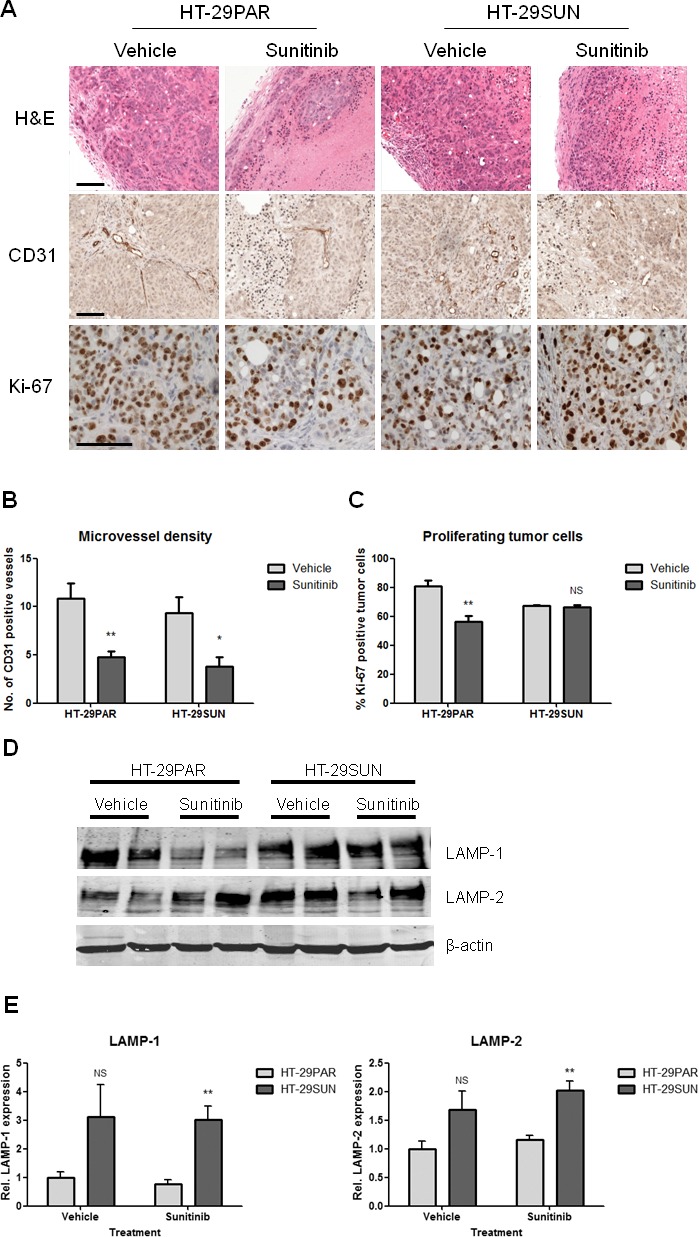
Immunohistochemical stainings and CD31, Ki-67 and LAMP-1 and -2 quantification (A) Representative pictures of (immunohistochemical) stainings of tumors established from HT-29 parental (HT-29PAR) and HT-29 sunitinib resistant (HT-29SUN) tumor cells, treated with vehicle or sunitinib. Upper panel: H&E staining; middle panel: CD31 staining; lower panel: Ki-67 staining. (B) Quantification of microvessel density (MVD) using CD31 staining (n = 8). Data are expressed as number of CD31 positive vessels per 200x field. (C) Quantification of tumor cell proliferation using Ki-67 staining (n = 4). Data are expressed as Ki-67 positive tumor cells as percentage of total tumor cells. (D) Western blot of lysosomal associated membrane proteins-1 and -2 (LAMP-1 and -2). Two representative tumor tissue samples per group are shown. (E) Quantification of LAMP-1 and -2 by western blot analysis (n = 4). LAMP-1 and -2 expression was corrected for β-actin expression, and normalized to vehicle-treated tumor samples. Results are shown as mean ± SEM; *, *P* < 0.05; **, *P* < 0.01, NS =not significant. Scale bars are 100 μm.

Representative examples of tumor tissue slices stained with H&E are shown in Figure 3*A* (upper panel). Sunitinib-treated HT-29PAR tumors revealed large areas of necrosis compared to its vehicle-treated tumors. No clear differences in viable or necrotic areas were observed between vehicle- and sunitinib-treated HT-29SUN tumors.

CD31 staining (representative pictures are shown in Figure 3*A*, middle panel) was used to quantify microvessel density (MVD), as a measure for angiogenesis inhibition. MVD was significantly reduced in both HT-29PAR and HT-29SUN tumors treated with sunitinib compared to vehicle-treated tumors (HT-29PAR: 4.7 ± 0.6 versus 10.9 ± 1.6 vessels per field (n = 4; *P* < 0.01); HT-29SUN: 3.8 ± 1.0 versus 9.3 ± 1.7 vessels per field (*P* < 0.05) for sunitinib and vehicle treatment, respectively (Figure 3*B*)). This corresponds to 56 ± 6% MVD inhibition in HT-29PAR and 59 ± 11% inhibition in HT-29SUN tumors after sunitinib treatment.

Representative pictures of Ki-67 staining as a measure of tumor cell proliferation are shown in Figure 3*A*, lower panel. Ki-67 quantification revealed a significant reduction in tumor cell proliferation by sunitinib treatment in HT-29PAR tumors by 30 ± 5% (81 ± 4% versus 56 ± 4% proliferating cells in PAR vehicle- versus sunitinib-treated mice; n = 4, *P* < 0.01; Figure 3*C*). In mice bearing HT-29SUN tumors, proliferation of tumors cells remained unaffected with a tumor cell proliferation of 67 ± 0% in vehicle- and 66 ± 1% in sunitinib-treated mice (*P* = ns).

Expression of lysosomal associated membrane proteins-1 and -2 (LAMP-1 and -2) was used as a measure of lysosomal capacity. Like *in vitro* [20], western blot analysis showed increased expression of both LAMP-1 and LAMP-2 in HT-29SUN tumors compared to HT-29PAR tumors (Figure 3*D*). Quantification demonstrated an increase in LAMP-1 expression in HT-29SUN versus HT-29PAR tumors of 3.1 fold in vehicle-treated (*P* = ns) and 3.9 fold in sunitinib-treated tumors (*P* < 0.01; Figure 3*E*). In addition, LAMP-2 expression was increased in HT-29SUN compared to HT-29PAR tumors with 1.7 fold in vehicle-treated (*P* = ns) and also 1.7 fold in sunitinib-treated tumors (*P* < 0.01).

### Chloroquine co-treatment

To determine the effect of chloroquine co-treatment with sunitinib, mice were treated with chloroquine diphosphate (50 mg/kg/day) starting seven days after tumor cell injection and continued for 30-40 days.

In HT-29PAR, neither single agent chloroquine treatment nor combination treatment with sunitinib did inhibit tumor growth compared to vehicle-treated tumors, with tumor weights of 0.51 ± 0.10 g versus 0.54 ± 0.04 g and 0.13 ± 0.01 g versus 0.11 ± 0.01 g, respectively (*P* = ns).

**Figure 4:**
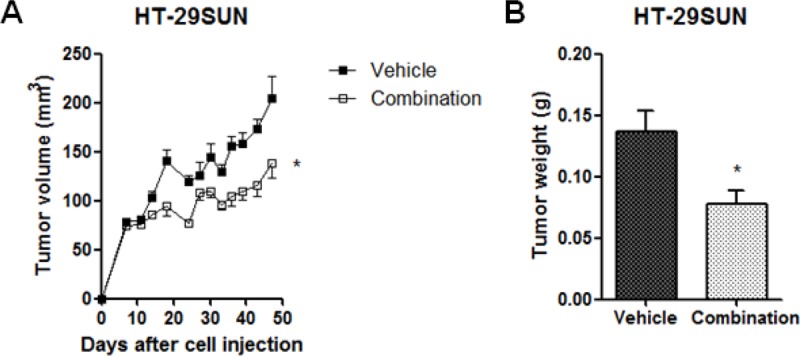
Co-treatment of sunitinib with chloroquine (A) Growth curves and (B) Tumor weights at the end of the experiment of tumors established from HT-29 sunitinib resistant (HT-29SUN) tumor cells after tumor cell injection. Mice were treated with vehicle or the combination of sunitinib malate (40 mg/kg/day) and chloroquine diphosphate (50 mg/kg/day). Results are shown as mean ± SEM (n = 4-6); *, *P* < 0.05.

In HT-29SUN, chloroquine as single agent did not inhibit tumor growth as well, with weights of 0.12 ± 0.02 g versus 0.14 ± 0.02 g for chloroquine versus vehicle treatment, respectively (*P* = ns). However, combination treatment of chloroquine and sunitinib did significantly inhibit HT-29SUN tumor growth with 32 ± 7% (mean ± SEM, n = 4-6; *P* < 0.05; Figure 4*A*) compared to vehicle. Similar, the weight of sunitinib/chloroquine-combination treated HT-29SUN tumors was lower than vehicle-treated tumors, with weights of 0.08 ± 0.01 g versus 0.14 ± 0.02 g (*P* < 0.05; Figure 4*B*).

## DISCUSSION

The development of acquired drug resistance is a major clinical problem for protein kinase inhibitors. We hypothesize that elucidating the mechanism of acquired resistance to sunitinib will provide new potential treatment strategies to circumvent or overcome this resistance. Therefore, we developed an *in vivo* tumor model established from HT-29 parental and sunitinib resistant tumor cells. We showed that treatment of mice with sunitinib did not inhibit tumor growth of HT-29SUN tumors while inhibiting tumor growth in HT-29PAR tumors significantly, indicating that tumor resistant factors play a larger role in resistance than host factors. This finding was supported by the fact that sunitinib concentrations in the resistant tumors were equal to parental tumors reflecting that differences in pharmacokinetic and drug delivery should not account for this difference. This observation may be of clinical relevance also based on our previously reported intratumoral sunitinib concentrations in patients that support a direct antitumor activity of sunitinib rather than solely inhibition of angiogenesis [20, 26].

Sunitinib is a multi-targeted tyrosine kinase inhibitor with anti-angiogenic properties by its potent VEGFR and/or PDGFR targeting. In preclinical studies sunitinib has been shown to significantly inhibit the growth of a number of colon cancer xenograft models such as HT-29, LS174t and Colo205 at doses of 40 mg/kg/day. However, an initial clinical phase II trial with sunitinib in patients with advanced colorectal cancer that was refractory to standard therapy demonstrated meaningful single-agent responses only in a subgroup of patients at the standard dosing of 50 mg/kg in 4-weeks-on, 2-weeks-off cycles (1 PR and 13 SD patients in a group of 84 patients [5].

The HT-29 colon cancer cell model is considered an appropriate model for studying sunitinib resistance mechanisms, because it is initially sensitive to conventional doses of 40 mg/kg sunitinib malate as used in mouse models by *Mendel et al*. [27]. Indeed, we confirmed a significant tumor growth inhibition of HT-29 tumors, either injected as cell suspension or as transplanted tumor pieces. Moreover, in another *in vivo* colon cancer model (Colo205) it was shown that after administration of a standard dose of 40 mg/kg sunitinib to the mice, a dose sufficient for target inhibition [27], gene expression changes were detected already 6 hours post-treatment [28]. In this *in vivo* model we observed a similar resistant phenotype of the tumors as we observed *in vitro* with a significant overexpression of the lysosomal compartment in the resistant tumors as measured by LAMP-1 and LAMP-2 overexpression. In addition, in the tumors established from the HT-29SUN cells, the effect of sunitinib treatment on microvessel density was similar to the effect in the HT-29PAR tumors, while the effect on the percentage of proliferating (Ki-67 positive) tumor cells was decreased in the sensitive, but not in the resistant HT-29SUN xenografts (Figure 3). Furthermore, we found a similar slower tumor growth rate of sunitinib resistant tumors compared to parental tumors as we previously observed *in vitro* [20]. The difference in growth rate between resistant and parental tumors indicates that the resistant tumors are significantly affected by continuous exposure to sunitinib. Whether this tumor resistant phenotype accurately predicts sunitinib resistance in patients needs further clinical evaluation. However, our results provide the first evidence that a tumor-cell-mediated mechanism of resistance may play a significant role in acquired resistance to sunitinib *in vivo*.

The intratumoral sunitinib uptake in HT-29 tumors was ~10 μM, which was very similar to the sunitinib tumor-uptake in patients treated with the standard dose of 50 mg daily [20]. We also found that tumor sunitinib uptake is not different in the sensitive and resistant tumors, suggesting that difference in exposure of the tumor compartment to sunitinib does not seem the cause of the resistant phenotype. Though an increased active cellular efflux of sunitinib might cause cellular resistance similar to resistance to cytotoxic agents and other protein kinase inhibitors [13;29], sunitinib is not a high affinity substrate for the most common ATP binding cassette transporters [30]. Moreover, we have shown that the cellular sunitinib levels are not decreased in HT-29 sunitinib resistant cells *in vitro.* Since sunitinib inhibits the vascularity in both tumor models to the same extent, the resistance phenotype does not seem a consequence to hypoxia-induced growth factor production in the tumors or mobilization of growth-factor producing myeloid cell populations [31] with consequent switch of the endothelial cell compartment from dependence on VEGFR signaling to other signaling pathways (i.e. EGF/EGFR or HGF/c-MET supported signaling) [16].

The observed lack of effect on tumor growth of sunitinib in the presence of an anti-angiogenic effect has been reported in a pancreatic ductal adenocarcinoma model as well [32]. Similar to this prior report, in our study the HT-29 sunitinib resistant tumors continued to grow independently from the vasculature nearby. In addition, we have no indication from exposure of human umbilical vein endothelial cells (HUVECs) to sunitinib *in vitro* that a significant down-regulation of CD31 expression is the reason for the apparent reduction of microvessels in the treated tumors (data not shown). Whether the slower growth rate of HT-29SUN tumors contributes to a more angiogenesis-independent tumor growth remains to be defined. In addition, treatment induced senescence may play a role in this angiogenesis independent growth, which may be mediated by p53/DEC1 activation and Raf-1/nuclear factor-(NF)-κB activation as shown by Zhu *et al*. [33].

The clinical significance of the HT-29 resistant phenotype remains to be investigated. It is important to consider that tumor-mediated rather than, or in addition to, a host-mediated resistance mechanisms might contribute to initial or acquired resistance to sunitinib or other VEGF receptor tyrosine kinase inhibitors.

As we previously reported that acidic lysosomes may be involved in the cause of resistance by intracellular sequestration of sunitinib due to its chemical characteristics [20], we found that LAMP-1 and -2 expression remains elevated in the resistant compared to the parental tumors. Due to the fact that sunitinib is hydrophobic (logP = 5.2), it can easily cross plasma membranes and other intracellular membranes. On the other hand, because it is a weak base (pKa = 8.95) [2], sunitinib will be increasingly protonated and thereby lose its ability to cross membranes in acidic environment. At a pH of 5, all sunitinib will be protonated. Therefore, upon entering an acidic organelle such as a lysosome (pH ≤ 5), sunitinib becomes protonated and cannot cross membranes anymore. Therefore, lysosomal sunitinib sequestration may play an important role in these *in vivo* experiments as a mediator of resistance. We have previously reported that short time exposure of bafilomycin A1, an inhibitor of the lysosomal function by increasing the lysosomal pH, does induce the release of sunitinib from lysosomes *in vitro* [20]. However, this agent is too toxic for *in vivo* experiments and therefore it is impossible to demonstrate whether it can revert sunitinib resistance *in vivo*. We examined chloroquine as an alternative for bafilomycin A1. In line with the recent report of Abdel-Aziz and co-workers, showing a potentiating effect of chloroquine on the antitumor activity of sunitinib [21], our current *in vivo* data indicate that systemic exposure to chloroquine, in combination with sunitinib, inhibits tumor growth of sunitinib resistant tumors in these xenografts. The activity of chloroquine to overcome sunitinib resistance in the HT-29SUN tumors may suggest that displacement of sunitinib from lysosomal stores may increase its general kinase inhibitory activity or for instance that the resistant tumor cells are more vulnerable to autophagy-inhibitory properties of chloroquine, which has been reported for several cell lines [21] and needs further clinical evaluation.

In conclusion, we show that acquired resistance to sunitinib is significantly dependent on tumor cells rather than host factors. Reduction in microvessel density was similar in sensitive and resistant tumors *in vivo*, indicating that in this model endothelial cell function/proliferation may play a secondary role in the resistance to sunitinib. The lysosomal capacity of tumor cells most likely contributes to sunitinib resistance. Based on these results, we started to evaluate the lysosomal function in resistance to sunitinib in a clinical trial in which patients with metastatic RCC are being rechallenged with sunitinib treatment following acquired resistance to sunitinib.

## MATERIAL AND METHODS

### Cell culture and reagents

The 786-O renal cell cancer cell line and the HT-29 colon cancer cell line were cultured in DMEM supplemented with 5% fetal bovine serum (FBS) and maintained in a humidified incubator containing 5% CO_2_ at 37°C. The cell lines originated from the American Tissue Culture Collection (ATCC) and were authenticated by STR profiling (Baseclear, Leiden, Netherlands). Resistance to sunitinib was induced as previously described [20]. Briefly, cells were continuously exposed to increasing concentrations of sunitinib. Resistance was evaluated by proliferation assays as previously described [20].

Sunitinib malate was kindly provided by Pfizer global Pharmaceuticals. For *in vitro* experiments, sunitinib malate was prepared as 20 mM stock solution in DMSO and stored at −20 °C. For *in vivo* experiments, sunitinib malate was dissolved at a concentration of 8 mg/ml in a vehicle containing distilled water with 1.8% NaCl, 0.5% carboxymethylcellulose, 0.4% tween80 and 0.9% benzylalcohol (pH adjusted to 6.0). The mixture was sonicated to achieve stable dispersion. Chloroquine (chloroquine diphosphate; Sigma-Aldrich) was prepared as a 10 mg/ml solution in PBS.

### *In vivo* mouse experiments

The animal research protocol was approved by the Institutional Animal Care and Use Committee at the Roswell Park Cancer Institute and was in accordance with the National Research Council's Guide for the Care and Use of Laboratory Animals. Four- to six-week-old male severe combined immunodeficient (SCID) mice were housed under pathogen-free conditions in a temperature-controlled room on a 12/12 hour light/dark schedule with food and water *ad libitum*.

Tumor establishment: 5×10^6^ parental or sunitinib resistant HT-29 cells grown under 10 μM sunitinib exposure were harvested from non-confluent monolayer cell cultures in 100 μL PBS. In the initial experiment tumor cells were injected subcutaneously, while in subsequent experiments equal viable tumor pieces from sacrificed mice were selected (approximately 1 mm^3^), and inoculated subcutaneously into a new cohort of mice (see below). Tumor growth was assessed twice weekly by caliper measurement, and size was expressed in mm^3^ using the standard formula: length × width^2^ × 0.52. Seven to twelve days post-injection/transplantation, tumor-bearing mice within either model were randomly distributed into 2 groups (6-8 animals per group) and treatment was started. Mice received treatment with sunitinib malate 40 mg/kg or a corresponding amount of vehicle, once daily, 7 days a week, by oral gavage. Mice selected for chloroquine treatment (n = 4), received 50 mg/kg chloroquine diphosphate by intraperitoneal (ip) injection, once daily, 4-8 h before sunitinib treatment. After 6-8 weeks of treatment, animals were sacrificed and blood was collected by cardiac puncture. Tumors were harvested and weighed, and tumor pieces were subsequently snap-frozen in liquid nitrogen.

In order to obtain tumor tissue for transplantation, three mice were bilaterally injected with either HT-29PAR or HT-29SUN cells and treated with vehicle or sunitinib, starting 7 days after injection and continued for 25 days. To establish parental tumors, tumor pieces of a vehicle-treated HT-29PAR tumor were transplanted in a new cohort of 16 mice. Since sunitinib resistance of HT-29SUN *in vitro* is transient [20], mice with HT-29SUN tumors planned for tumor transplantation were treated with sunitinib, in order to maintain resistance. For selection of a more rapid tumor growth rate, the fastest growing tumor was transplanted in a new cohort of 16 mice.

### Immunohistochemistry

Tumor pieces were fixed in 10% formalin and embedded in paraffin. Deparaffinization and rehydration of 3-μm-thick sections were followed by H&E or specific immunohistochemical staining. After blocking, sections were incubated with primary antibodies to detect CD31 (Santa Cruz Biotechnology), Ki-67 (Thermo Scientific) or the appropriate IgG controls, followed by incubation with secondary antibodies. Staining was developed by incubation with 3,3′-diaminobenzidine, and counterstained with hematoxylin. Subsequently sections were dehydrated in alcohol and xylene and mounted. Images were captured using an Olympus microscope (model BX50F) with a Leica camera (DC300 V2.0) and Leica software.

Microvessel density (MVD; n = 8 per group) was scored using CD31 staining. Viable areas of the tumor were selected, and MVD was subsequently assessed in 5 randomly selected fields (200x). The results are shown as number of vessels/field. Ki-67 quantification (n = 4 per group) was performed using a Leica microscope (DMLM) with a Leica CCD camera and Leica QPRODIT software (version V3.2). At first, viable areas of the tumor were selected using a 2.5x objective. Over this measurement area, 600 fields of vision were randomly equidistantly placed by the QPRODIT software. At 40x objective, each field of vision was investigated using a 6-point electronic grid system of three ‘Weibel-type’ test lines (grid point distance was 70.0 μm) of which the center grid point was registered. A point falling on a tumor cell was counted, but other tissue or empty space (stroma, infiltrate, scar, no tissue) was ignored. When the center point was scored (as Ki-67 negative or positive tumor cell) or ignored in one field of vision, the next field was selected using a random systematic sampling approach in the measurement area. After the measurements, the QPRODIT software calculated automatically the percentage of Ki-67 negative and positive tumor cells (as a percentage of total counted tumor cells).

### Western blot analysis

Lysates (n = 4 per group) from snap-frozen tumor tissues were prepared using M-PER lysis buffer containing phosphatase and protease inhibitors (Thermo Scientific) added to 20 μm cryoslides. Tissues were incubated with the lysis buffer mixture for 20 minutes on ice and subsequently centrifuged (10,000 x g) for 15 minutes at 4 °C. Supernatant was collected and stored at −80 °C until analysis. Protein concentrations were determined with Micro BCA protein assay (Thermo Scientific). Samples containing 20 μg protein underwent electrophoresis on 10% SDS polyacrylamide gels and were subsequently transferred to PVDF membranes (Immobilon-FL, Millipore). Proteins were detected using LAMP-1 (sc-5570) and LAMP-2 (sc-18822) antibodies (Santa Cruz biotechnology) and β-actin (Sigma-Aldrich). After incubation with IRDye infrared dye labeled secondary antibodies (LI-COR Biosciences), membranes were scanned with the Odyssey Infrared Imaging System (LI-COR Biosciences). LAMP-1 and -2 expression was analyzed with the accompanying software program (LI-COR Biosciences), and subsequently corrected for β-actin expression and normalized to vehicle-treated tumor samples. Western blots were performed twice.

### Sunitinib measurements by LC-MS/MS

Tumor and skin pieces as well as serum (n = 8 per group) were analyzed for sunitinib amount by a validated liquid chromatography - tandem mass spectrometry (LC-MS/MS) assay, as reported previously [20;25]. Briefly, at the end of the mice experiments, blood was drawn by cardiac puncture and transferred to 1 ml tubes. Tubes were inverted 5 times and stored at RT for 1 hour. After centrifugation for 10 minutes at 13,000 rpm RT, the supernatant was transferred into a new tube and stored at −80°C. Serum was analyzed by LC-MS/MS as previously reported [25]. Tumor and skin pieces, after harvesting, were immediately snap-frozen in liquid nitrogen and stored at −80°C until analyzed. For analysis, tissue pieces were weighed frozen and suspended in milliQ water (200 μl) and subsequently freeze-dried. Lyophilized tumor and skin tissue were subsequently resuspended in acetonitrile:water (ACN:H_2_O; 5:1), homogenized and briefly sonicated (30 sec). After centrifugation, supernatant was taken for LC-MS/MS analysis [25]. Data are expressed in micromolar (μM) and are conversed as previously described [20].

### Statistical analysis

Data are expressed as mean ± standard error of the mean (SEM) or, when indicated, as median with range. When appropriate, results are shown as normalized data. Statistical analyses were performed using Student's *t-*test. A *P* value < 0.05 was considered to be statistically significant. *, *P* value < 0.05; **, *P* value < 0.01; ***, *P* value < 0.001.

## SUPPLEMENTARY MATERIAL FIGURE



## References

[1] Garraway LA, Janne PA (2012). Circumventing cancer drug resistance in the era of personalized medicine. Cancer Discov.

[2] Goodman VL, Rock EP, Dagher R, Ramchandani RP, Abraham S, Gobburu JV, Booth BP, Verbois SL, Morse DE, Liang CY, Chidambaram N, Jiang JX (2007). Approval summary: sunitinib for the treatment of imatinib refractory or intolerant gastrointestinal stromal tumors and advanced renal cell carcinoma. Clin Cancer Res.

[3] Raymond E, Dahan L, Raoul JL, Bang YJ, Borbath I, Lombard-Bohas C, Valle J, Metrakos P, Smith D, Vinik A, Chen JS, Hörsch D, Hammel P (2011). Sunitinib malate for the treatment of pancreatic neuroendocrine tumors. N Engl J Med.

[4] Motzer RJ, Hutson TE, Tomczak P, Michaelson MD, Bukowski RM, Oudard S, Negrier S, Szczylik C, Pili R, Bjarnason GA, Garcia-del-Muro X, Sosman JA, Solska E (2009). Overall survival and updated results for sunitinib compared with interferon alfa in patients with metastatic renal cell carcinoma. J Clin Oncol.

[5] Saltz LB, Rosen LS, Marshall JL, Belt RJ, Hurwitz HI, Eckhardt SG, Bergsland EK, Haller DG, Lockhart AC, Rocha Lima CM, Huang X, DePrimo SE, Chow-Maneval E (2007). Phase II trial of sunitinib in patients with metastatic colorectal cancer after failure of standard therapy. J Clin Oncol.

[6] Faivre S, Demetri G, Sargent W, Raymond E (2007). Molecular basis for sunitinib efficacy and future clinical development. Nat Rev Drug Discov.

[7] Fabian MA, Biggs WH 3rd, Treiber DK, Atteridge CE, Azimioara MD, Benedetti MG, Carter TA, Ciceri P, Edeen PT, Floyd M, Ford JM, Galvin M, Gerlach JL (2005). A small molecule-kinase interaction map for clinical kinase inhibitors. Nat Biotechnol.

[8] Karaman MW, Herrgard S, Treiber DK, Gallant P, Atteridge CE, Campbell BT, Chan KW, Ciceri P, Davis MI, Edeen PT, Faraoni R, Floyd M, Hunt JP (2008). A quantitative analysis of kinase inhibitor selectivity. Nat Biotechnol.

[9] Bottsford-Miller JN, Coleman RL, Sood AK (2012). Resistance and escape from antiangiogenesis therapy: clinical implications and future strategies. J Clin Oncol.

[10] Hammers HJ, Verheul HM, Salumbides B, Sharma R, Rudek M, Jaspers J, Shah P, Ellis L, Shen L, Paesante S, Dykema K, Furge K, Teh BT (2010). Reversible epithelial to mesenchymal transition and acquired resistance to sunitinib in patients with renal cell carcinoma: evidence from a xenograft study. Mol Cancer Ther.

[11] Bergers G, Hanahan D (2008). Modes of resistance to anti-angiogenic therapy. Nat Rev Cancer.

[12] Ellis LM, Hicklin DJ (2008). Pathways mediating resistance to vascular endothelial growth factor-targeted therapy. Clin Cancer Res.

[13] Broxterman HJ, Gotink KJ, Verheul HM (2009). Understanding the causes of multidrug resistance in cancer: a comparison of doxorubicin and sunitinib. Drug Resist Updat.

[14] Tang SC, Lankheet NA, Poller B, Wagenaar E, Beijnen JH, Schinkel AH (2012). P-glycoprotein (ABCB1) and breast cancer resistance protein (ABCG2) restrict brain accumulation of the active sunitinib metabolite N-desethyl sunitinib. J Pharmacol Exp Ther.

[15] Huang D, Ding Y, Zhou M, Rini BI, Petillo D, Qian CN, Kahnoski R, Futreal PA, Furge KA, Teh BT (2010). Interleukin-8 mediates resistance to antiangiogenic agent sunitinib in renal cell carcinoma. Cancer Res.

[16] Shojaei F, Lee JH, Simmons BH, Wong A, Esparza CO, Plumlee PA, Feng J, Stewart AE, Hu-Lowe DD, Christensen JG (2010). HGF/c-Met acts as an alternative angiogenic pathway in sunitinib-resistant tumors. Cancer Res.

[17] Su SC, Hu X, Kenney PA, Merrill MM, Babaian KN, Zhang XY, Maity T, Yang SF, Lin X, Wood CG (2013). Autotaxin-lysophosphatidic Acid signaling axis mediates tumorigenesis and development of acquired resistance to sunitinib in renal cell carcinoma. Clin Cancer Res.

[18] Yang J, Ikezoe T, Nishioka C, Takezaki Y, Hanazaki K, Taguchi T, Yokoyama Al (2012). Long-term exposure of gastrointestinal stromal tumor cells to sunitinib induces epigenetic silencing of the PTEN gene. Int J Cancer.

[19] Sato M, Nakai Y, Nakata W, Yoshida T, Hatano K, Kawashima A, Fujita K, Uemura M, Takayama H, Nonomura N (2013). EMMPRIN promotes angiogenesis, proliferation, invasion and resistance to sunitinib in renal cell carcinoma, and its level predicts patient outcome. PLoS One.

[20] Gotink KJ, Broxterman HJ, Labots M, de Haas RR, Dekker H, Honeywell RJ, Rudek MA, Beerepoot LV, Musters RJ, Jansen G, Griffioen AW, Assaraf YG, Pili R (2011). Lysosomal sequestration of sunitinib: a novel mechanism of drug resistance. Clin Cancer Res.

[21] Abdel-Aziz AK, Shouman S, El-Demerdash E, Elgendy M, Abdel-Naim AB (2014). Chloroquine synergizes sunitinib cytotoxicity via modulating autophagic, apoptotic and angiogenic machineries. Chem Biol Interact.

[22] Slater AF (1993). Chloroquine: mechanism of drug action and resistance in Plasmodium falciparum. Pharmacol Ther.

[23] Savarino A, Boelaert JR, Cassone A, Majori G, Cauda R (2003). Effects of chloroquine on viral infections: an old drug against today's diseases?. Lancet Infect Dis.

[24] Zhao H, Cai Y, Santi S, Lafrenie R, Lee H (2005). Chloroquine-mediated radiosensitization is due to the destabilization of the lysosomal membrane and subsequent induction of cell death by necrosis. Radiat Res.

[25] Honeywell R, Yarzadah K, Giovannetti E, Losekoot N, Smit EF, Walraven M, Lind JS, Tibaldi C, Verheul HM, Peters GJ (2010). Simple and selective method for the determination of various tyrosine kinase inhibitors used in the clinical setting by liquid chromatography tandem mass spectrometry. J Chromatogr B Analyt Technol Biomed Life Sci.

[26] Labots M, Neerincx M, Van der Mijn JC, Dekker H, Honeywell RJ, Rovithi MN, Gotink KJ, Voebel-De Jong M, Van der Peet DL, Meijerink MR, Meijer GA, Jimenez CR, Peters GJ (2013). Tumor, skin, and plasma concentrations of protein kinase inhibitors (PKIs) in patients with advanced cancer. J Clin Oncol.

[27] Mendel DB, Laird AD, Xin X, Louie SG, Christensen JG, Li G, Schreck RE, Abrams TJ, Ngai TJ, Lee LB, Murray LJ, Carver J, Chan E (2003). *In vivo* antitumor activity of SU11248, a novel tyrosine kinase inhibitor targeting vascular endothelial growth factor and platelet-derived growth factor receptors: determination of a pharmacokinetic/pharmacodynamic relationship. Clin Cancer Res.

[28] Morimoto AM, Tan N, West K, McArthur G, Toner GC, Manning WC, Smolich BD, Cherrington JM (2004). Gene expression profiling of human colon xenograft tumors following treatment with SU11248, a multitargeted tyrosine kinase inhibitor. Oncogene.

[29] Broxterman HJ, Lankelma J, Hoekman K (2003). Resistance to cytotoxic and anti-angiogenic anticancer agents: similarities and differences. Drug Resist Updat.

[30] Hu S, Chen Z, Franke R, Orwick S, Zhao M, Rudek MA, Sparreboom A, Baker SD (2009). Interaction of the multikinase inhibitors sorafenib and sunitinib with solute carriers and ATP-binding cassette transporters. Clin Cancer Res.

[31] Shojaei F, Wu X, Qu X, Kowanetz M, Yu L, Tan M, Meng YG, Ferrara N (2009). G-CSF-initiated myeloid cell mobilization and angiogenesis mediate tumor refractoriness to anti-VEGF therapy in mouse models. Proc Natl Acad Sci U S A.

[32] Olson P, Chu GC, Perry SR, Nolan-Stevaux O, Hanahan D (2011). Imaging guided trials of the angiogenesis inhibitor sunitinib in mouse models predict efficacy in pancreatic neuroendocrine but not ductal carcinoma. Proc Natl Acad Sci U S A.

[33] Zhu Y, Xu L, Zhang J, Hu X, Liu Y, Yin H, Lv T, Zhang H, Liu L, An H, Liu H, Xu J, Lin Z (2013). Sunitinib induces cellular senescence via p53/Dec1 activation in renal cell carcinoma cells. Cancer Sci.

