# Supplementary material for: Acquired tumor cell resistance to sunitinib causes resistance in a HT-29 human colon cancer xenograft mouse model without affecting sunitinib biodistribution or the tumor microvasculature
Source: Oncoscience. 2014 Dec 15;1(12):844–53. doi: 10.18632/oncoscience.106 (PMC4303892; doi:10.18632/oncoscience.106)
Supplement: Supplementary file 1 [file oncoscience-01-0844-s001.pdf]

Acquired tumor cell resistance to sunitinib causes resistance in a HT29 human colon cancer xenograft mouse model without affecting sunitinib biodistribution or the tumor microvasculature

Supplementary Material

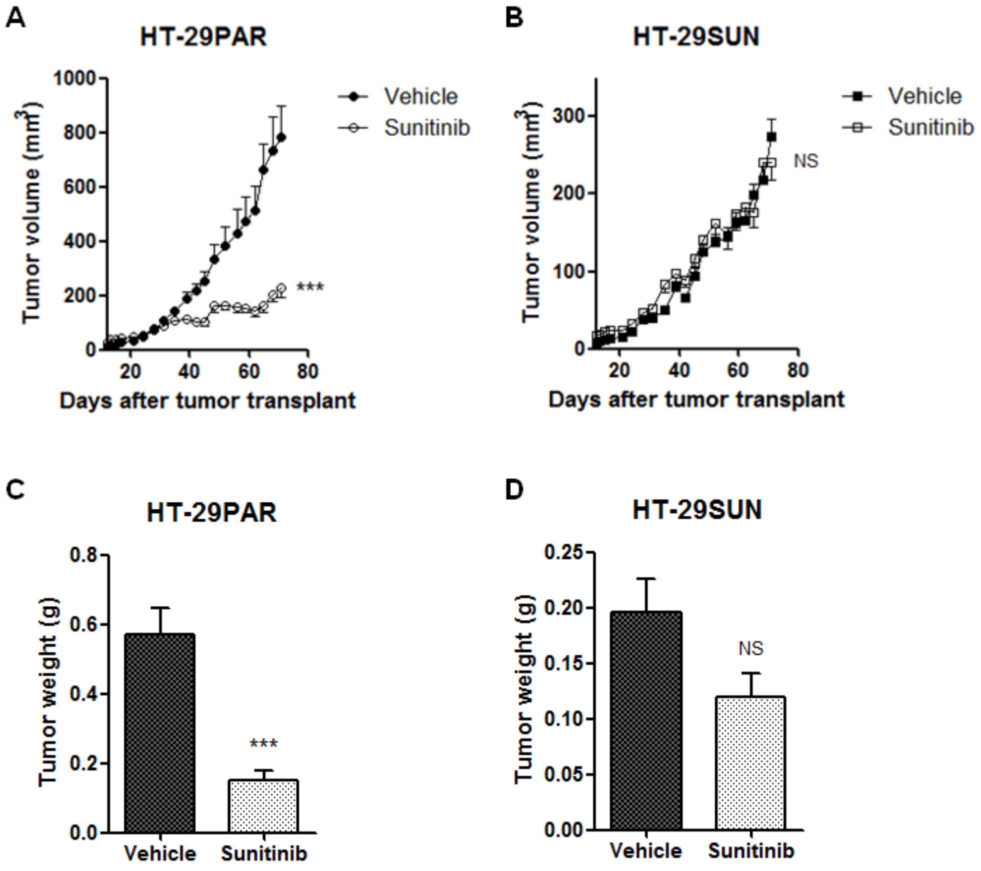

Supplementary Figure S1: Sunitinib treatment of HT-29PAR and HT-29SUN tumors after tumor transplant. (A) and (B), Growth curves of tumors established from HT-29 parental (HT-29PAR; A) and HT-29 sunitinib-resistant (HT-29SUN; B) tumor cells after tumor transplantation, treated with vehicle or sunitinib (40 mg/kg/day). (C) and (D), Tumor weights at the end of the experiment of HT-29PAR (C) and HT-29SUN (D) tumors. Results are shown as mean ± SEM (n = 8); ***, $P < 0.001$.
